# Mad River Dental Society

**Published:** 1867-09

**Authors:** 


### MAD RIVER DENTAL SOCIETY.

THE regular quarterly meeting of this society was held on the 3d inst., at Springfield, Ohio. There was a good attendance of the membership. A very interesting programme of subjects was presented for consideration and discussion, into which the majority of the members entered with spirit and earnestness, and thus demonstrated that it truly lives and possesses a power for good within the sphere of its influence, not excelled by any other society. It is truly gratifying to observe the progress demonstrated by a comparison of the subjects now presented for discussion and those of former times, as well as the character of the discussions. Association has been one of the chief instrumentalities in the elevation, growth and development of our profession. The work accomplished by this agency cannot be performed by any other.

There is a grand indication in the fact that new societies are being organized more rapidly than ever before.

